# Bioprocessing by Decellularized Scaffold Biomaterials in Cultured Meat: A Review

**DOI:** 10.3390/bioengineering9120787

**Published:** 2022-12-09

**Authors:** Hongyun Lu, Keqin Ying, Ying Shi, Donghong Liu, Qihe Chen

**Affiliations:** 1 Department of Food Science and Nutrition, Zhejiang University, Hangzhou 310058, China; 2 College of Food Science and Technology, Nanjing University, Nanjing 210095, China; 3 Innovation Center of Yangtze River Delta, Zhejiang University, Jiashan 310000, China

**Keywords:** cell-cultured meat, biomaterial, cellular agriculture, decellularized scaffolds, future food, decellularization

## Abstract

As novel carrier biomaterials, decellularized scaffolds have promising potential in the development of cellular agriculture and edible cell-cultured meat applications. Decellularized scaffold biomaterials have characteristics of high biocompatibility, bio-degradation, biological safety and various bioactivities, which could potentially compensate for the shortcomings of synthetic bio-scaffold materials. They can provide suitable microstructure and mechanical support for cell adhesion, differentiation and proliferation. To our best knowledge, the preparation and application of plant and animal decellularized scaffolds have not been summarized. Herein, a comprehensive presentation of the principles, preparation methods and application progress of animal-derived and plant-derived decellularized scaffolds has been reported in detail. Additionally, their application in the culture of skeletal muscle, fat and connective tissue, which constitute the main components of edible cultured meat, have also been generally discussed. We also illustrate the potential applications and prospects of decellularized scaffold materials in future foods. This review of cultured meat and decellularized scaffold biomaterials provides new insight and great potential research prospects in food application and cellular agriculture.

## 1. Introduction

In the context of rapid population growth and economic development, there is a growing demand for and reliance on animal-derived protein. With the increasing demand for meat worldwide of more than 70%, resources on the earth will not provide enough meat to the world’s population by 2050 [1]. In order to replace the supply of animal meat and avoid the impact of livestock farming on soil destruction, water resources pollution and greenhouse gas emissions [2], it is essential to explore new sustainable production methods of eatable protein. Currently, synthesized meat is divided into two main types. The first one is meat analogs derived from plant and insect protein, and it uses protein-modified plant material to simulate the taste of meat and provide more sources of protein from insects [3]. However, due to the difference in nutritional value and meat flavor, this method cannot completely replace the current meat market. The second is cultured meat produced by in vitro proliferation and differentiation of animal stem cells [4], which has shown great potential and become an unstoppable trend for application in developing new meat resources. Laboratory-grown meat, also called cultured meat, clean meat, synthetic meat or in vitro meat [5], is a foodstuff formed by the proliferation of animal cells in vitro, and is mainly composed of skeletal muscle, fat and connective tissue [6]. In fact, the development of technologies such as stem cell isolation and identification, cell culture, and tissue engineering have gradually made it possible to culture meat in vitro. 

Most mammalian cells are anchorage-dependent, which leads them to adhere to the surface of vector in cell proliferation. Previously, microcarriers were usually chemically synthesized polymers, which were manufactured on a large scale. However, related applications in cell expansion are relatively limited due to the lack of cell recognition sites [7]. Currently, microcarriers have the advantage of higher efficiency in material transport, leading to the developed culture system of adherent cells. On the other hand, micro-carriers have been used to amplify specific cells to produce monoclonal antibodies, proteins, vaccines, etc. A variety of research studies are focused on their potential applications in the field of regenerative medicine and tissue engineering. In fact, the simplest scaffolds are microcarriers, but mostly they play a role only during the suspension culture [8], thus the application of microcarriers in meat production has not been extensively studied [9]. To obtain cultured meat with a more realistic structure and texture, a scaffold that provides vascularization should be explored [8,10]. Generally speaking, cultured meat is produced by proliferating and adhering muscle cells to a carrier, then transferred to a bioreactor with a growth medium for culture. Cell-supporting scaffolds are intended to resemble the extracellular matrix (ECM) in structure and to some extent in biochemical properties that support cell structure and function in natural tissues [11]. Therefore, it is essential to find a suitable scaffold during the production of meat that meets different structural demands [12].

To this end, we systematically summarize the superior types of scaffolds, emphasizing the production methods and application directions in cell-culture meat, to provide a basis for the development and growth of cell-cultured meat.

## 2. Review Methodology

The studies published in the last five years were collected from several databases including Web of Science and PubMed with the keywords of ‘cell-cultured meat’, ‘biomaterial’, ‘cellular agriculture’ and ‘decellularized scaffolds’. After removing conference proceedings, letters, abstracts and unrelated topics, highly related references were cited.

## 3. Challenges of Cell-Cultured Meat Bioprocessing

Cell-culture meat is currently one of the most cutting-edge research directions and important future trends in the development of cellular agriculture. While mimicking the taste, texture and appearance of meat, cell-culture products have a positive effect in ensuring food quality and safety, maintaining sustainable development of resources, avoiding foodborne diseases and reducing animal suffering [13]. However, cultured meat production is currently more expensive than comparable conventional meat products. Currently, cell culture meat is facing many important challenges for large-scale commercial production. Therefore, research must carefully consider not only the development of functional immortalized cell line, optimization of cell culture medium but also the design of cell bioreactors and biomaterials, as well as development of safe and economical edible scaffolds and other related challenges [1]. In the field of cultured meat research, myoblast cells, satellite cells, embryonic stem cells and adipose tissue-derived stem cells are attracting more and more attention [14], because they are expected to develop into skeletal muscle tubes, mature muscle fibers and fatty tissue. However, there is still an urgent need to address the problem of decreased cell expansion capacity during cell culture to improve its differentiation and proliferation capacity for industrial-scale meat production applications. Additionally, cultured meat for the food industry requires large-scale cultivation, low resource consumption and the short production time of cell culture. Thus, it is important to explore the improvement and optimization of multiple cell culture technologies including microcarriers, suspension cultures, packed bed bioreactors, etc. [15], so as to achieve efficient and large-scale production of cultured meat.

Scaffold biomaterials serve as an integrated support network that is a key component of cellular agriculture, with cells expanding and differentiating in an anchorage-dependent manner. Scaffolds for cell culture meat are usually biodegradable, but if not, they must be safe, economical and easy to produce on a large scale. This porous network allows the flow of oxygen and nutrients and removal of waste products to maintain cellular metabolic functions and avoid the formation of necrotic cores. However, although simple scaffolds with medium infusion can grow muscle fibers, only in combination with tissue technology can they produce structured products other than minced meat [16]. For this reason, the production of cultured meat requires scaffolds with the ability to support the differentiation of multiple cell types and their co-cultures, and overcome the thickness limitation of the cultured meat tissue. This is a challenge for the production of cell-cultured meat and an opportunity to drive further development of the scaffold [8]. There is evidence that culturing multiple cell types (e.g., myogenic cells) and at least one type of ECM-secreting cell on a porous scaffold and spreading them on the scaffold can eventually lead to the formation of muscle cell fibers and cell-cultured meat [17].

### 3.1. Current Research Progress on Scaffolds

Currently, the widely studied scaffold is a type of three-dimensional (3D) structure with porous characteristics. In general, it mimics the extracellular matrix and is therefore capable of prompting the attachment, proliferation, and differentiation of essential skeletal muscle, adipose and connective tissue cells. The ideal scaffold should have a large growth and attachment surface area, be flexible to allow for shrinkage, maximize medium diffusion and be easily separated from the meat culture. The manufacturing technologies for scaffolds are quite diverse, including electrospinning, casting mold systems, injectable systems and extrusion-based 3D printing, but there exist corresponding disadvantages [18], such as uncontrolled size, poor reproducibility, chemical residues, difficulties in integrating vascular networks and insufficient interconnectivity [19]. Therefore, more suitable technologies still need to be explored in further research.

### 3.2. Applied Types of Meat Scaffolds in Cell Culture

Typically, the scaffolds are divided into natural polymer scaffolds and synthetic scaffolds. Among them, natural polymer scaffolds, such as animal-derived collagen [20], gelatin [21], arthropods and fungi-derived chitin [22], are demonstrated to be ideal for making scaffolds with ECM-like characteristics. However, due to their high cost, collagen scaffolds remain difficult to produce, which limit their large-scale application in the cell-cultured meat industry. Therefore, scaffold materials synthesized from food materials, such as tapioca starch, cellulose, alginate, gelatin and agarose [2,10,23,24], have been widely reported to support cell growth and thus have promising applications in cell culture meat. However, these synthetic scaffold materials are less biocompatible, and it is difficult to ensure that cell culture meat has a similar muscle structure and morphological texture to natural meat. The natural tissue morphology of the cell culture meat was generally determined by the scaffold material. Therefore, the morphology of the scaffold material has attracted attention. Orellana et al. [25] then produced films with parallel microchannel structures to induce cells to align as muscle fibers, thus making it possible to culture in vitro cell culture meat with natural structures. The feasibility of this method for in vitro meat production was also confirmed by Acevedo et al. [26], due to the fact that scaffolds with a transverse texture structure facilitate the formation of myotubes because of their similarity to muscle structure [27]. In this aspect, natural material scaffolds may be more appropriate due to their unique structure. Additionally, through a combination of physical, chemical and biological methods, a wide range of natural tissues of animals, plants and microorganisms can be prepared as decellularized scaffolds. Such a new decellularized scaffold has been proposed as a scaffold material due to its complete structure and biocompatibility. Therefore, over the past decades, the materials of scaffolds have taken a giant leap from simple collagen and alginate to complex decellularized scaffold biomaterials [28]. 

Typically, scaffolds for food and cellular agriculture are required to be degradable, or safe and palatable [1]. In fact, the most worrisome potential hazard of scaffolds and microcarriers during the whole process of cell-cultured meat production is the harmful substances in the material, which means the material itself and its degradation products may not be safe for consumption [29]. Decellularized scaffolds are potential ideal scaffold biomaterials for cellular agriculture or tissue engineering. Such scaffold biomaterials are derived from natural tissues and therefore are considered safe and green, and mostly edible or degradable. Moreover, they have high biocompatibility, bioactivity, and can provide highly vascularized and suitable microstructure and mechanical support, showing great potential research value and application prospects. The biological tissue environment is not a simple superposition of proteins, so it is difficult for 3D-printed scaffolds to accurately mimic complex tissue culture environments [30]. In contrast, decellularized scaffolds can truly solve this challenge. A large number of plant tissues even have been shown to support the growth of a wide range of mammalian cell types [31] (Table 1).

**Figure 1:**
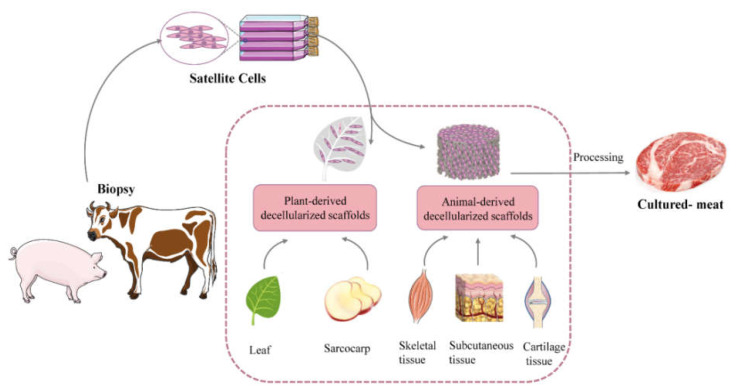
Schematic of cultured meat produced from decellularized scaffolds.

Decellularized scaffolds have been widely used in the medical industry, and further their development promoted in the food industry, which is summarized in Table 1. Herein, we comprehensively review the origin, applications, principles, classification and characteristics of decellularized scaffolds, a natural scaffold, to provide a theoretical reference and application basis for research related to cultured meat production (Figure 1). 

## 4. The Principles, Methods, and Application of Animal-Derived Decellularized Scaffolds

Animal decellularized scaffolds have played an important role in drug screening, allogeneic organ transplantation [55], regenerative medicine and tissue engineering, which also shows their great potential in meat production. Currently, animal-derived scaffolds have proven more suitable for the growth of myogenic cells because they are closer to the natural physiological properties of the cell growth environment, whereas synthetic biomaterials cannot be used to achieve tissue contraction [56]. Therefore, animal-derived decellularized scaffolds have a definite advantage over non-animal derived scaffold biomaterials in cultured meat and tissue engineering.

### 4.1. Application Principles of Animal-Derived Decellularized Scaffolds

After the decellularization process of animal tissues, cellular components, such as nuclear components and cytoplasm, are effectively removed, so that the decellularized scaffolds will not cause immune rejection and their biocompatibility is greatly improved [57]. The ECM has a dynamic, highly complex molecular structure that is rich in multiple components. These components can interact with each other and determine the form and function of the tissue [58]. While the cellular components are removed, most of the ECM components in the biomaterials are retained. These natural ECMs are rich in bioactive substances such as growth factors and cytokines, which can regulate the behaviors of certain cells, provide them with an appropriate growth microenvironment, and preserve the stem cell niche of natural tissues and regenerative capacity in vivo [59]. Therefore, decellularized scaffolds have strong bioactivity and the ability to induce cellular behaviors.

Most of the animal-derived decellularized scaffolds have specific microarchitectures and mechanical characterization, thus they can provide mechanical support for adherent cell adhesion and growth. Additionally, as a biological material for cultured meat production, the scaffold should not affect the edibility of the meat. Therefore, it is expected to be separated from the cell cultures or degraded at some stage. It would have more production advantages if the scaffold itself was edible and could be fully embedded in the meat without affecting its taste or sensory properties [9].

### 4.2. Preparation Methods of Animal-Derived Decellularized Scaffolds

The methods for preparation of animal-derived decellularized scaffolds, as a biomaterial used to produce edible meat, still have more space for exploration and improvement. In addition to the replacement ability of regenerative medicine, decellularized scaffolds for food meat production should meet the requirements of larger-scale production and lower cost, while their purity does not have to be as high as medical supplies [56]. Therefore, searching for the most appropriate methods for the preparation of animal-derived decellularized scaffolds and optimal culture conditions holds the key to the puzzle of cultured meat.

**Figure 2:**
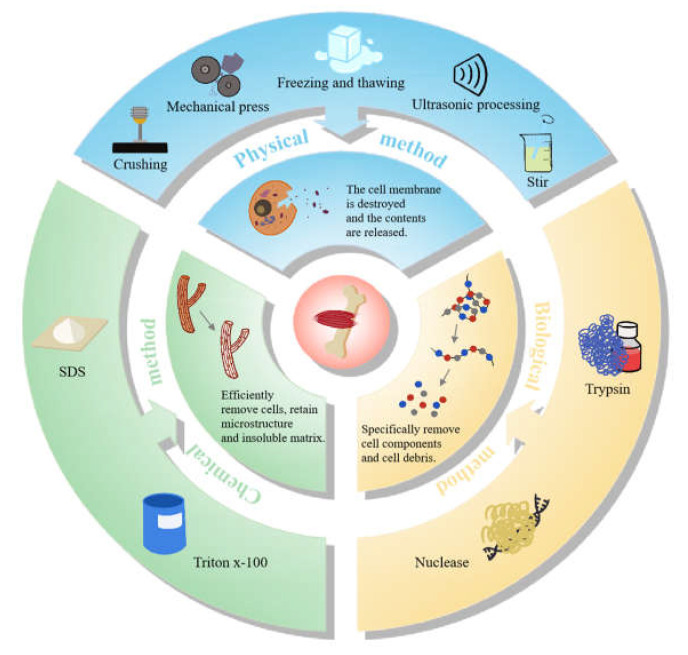
The methods for the preparation of animal-derived decellularized scaffolds.

The methods of decellularization are mainly divided into physical methods, chemical methods, biological methods, and a combination of the above three treatments. Taken together, the common methods for preparation are summarized as demonstrated in Figure 2. Usually, physical methods, including crushing, stirring, ultrasonic processing, mechanical press, freezing and thawing, etc., can disrupt the cell membrane and release the contents. Due to the poor ability to decellularize the tissue alone, physical methods are mostly used in combination with other treatments [60]. After that, animal tissues should be further treated with chemical means such as sodium dodecyl sulfate (SDS) and Triton X-100, and biological means such as nuclease and trypsin, to disrupt the interactions among DNA, proteins and lipids. Meanwhile, this joint approach also eliminated cellular debris and promoted detergent infiltration [61], so that the cellular components can be more efficiently eliminated. The decellularization can be evaluated by characterization such as hematoxylin-eosin (HE) staining and DNA quantification, and the mechanical properties can be measured by nanoindentation and uniaxial compression testing, while scanning electron microscope (SEM) analysis, micro-CT and other techniques can be used to exhibit the structural properties of the scaffolds [62] (Figure 2). 

However, the precision and accuracy of decellularization is not completely tested. The mechanical properties and degradation rate of the decellularized scaffolds may not be sufficient for industry [63]. Therefore, a more convenient and efficient method of decellularization is worth exploring. 

### 4.3. The Application of Animal-Derived Decellularized Scaffolds of Cultured Meat

The preparation of animal-derived decellularized scaffolds has been extensively studied and used in practice, and they are widely recognized for their value as promising, tissue-engineered biomaterials, especially for the production of allogeneic animal tissues with homologous decellularized scaffolds. Wang et al. [41] selected tendons to prepare decellularized scaffolds, and the tendon ECMs showed striking similarity to natural tendons in bioactive components, collagen arrangement and biomechanical characteristics. Lin et al. [33] concluded that both decellularized porcine subcutaneous and visceral adipose tissue have a positive effect on the in vitro culture, morphological changes and differentiation of human adipose-derived stem cells. Sun et al. [36] compared decellularized ECMs derived from cartilage tissues to those derived from chondrocytes or stem cells, finding that the former was more likely to promote chondrogenic differentiation, while the latter contributes to cell proliferation and thus supports chondrogenesis.

Meanwhile, animal-derived decellularized scaffolds were extracted from single tissue in vitro culture, and their biomechanical characteristics were completely different [64]. Furthermore, Hanai et al. [32] completed the decellularization process of porcine tissues including cartilage, meniscus, ligament, tendon, muscle, synovium, fat pad, fat and bone, and found that the decellularized ECMs derived from different tissues contained different specific components, such as hydroxyproline, sulfated glycosaminoglycan and growth factors, which had different specific differentiation potential of skeletal tissues in cartilage, ligament and bone-derived ECMs. Because of the structural and functional similarity of ECMs between marine tissues and mammalian tissues, Lau et al. [65] prepared decellularized tilapia skin scaffolds, which have filled the gaps in commercially available non-mammalian decellularized scaffold products. The collagen of tilapia skin has a high denaturation temperature, so it is suitable for use as a biological material. Khajavi et al. [37] successfully promoted cartilage matrix synthesis through decellularized sturgeon fish cartilage. Adipose tissue contains a variety of ECM components including collagen, elastin, and biological macromolecules; it has significant potential for chondrogenic differentiation. Thus, Ibsirlioglu and Elçin [34] used adipose tissues for the preparation of decellularized scaffolds to promote chondrocyte adhesion, proliferation and differentiation. Various innovative studies, such as decellularized bovine intervertebral disc scaffolds supporting in vitro culture of multiple xenogeneic cells [66] and decellularized fish scale scaffolds for bone tissue engineering [35], have also demonstrated the increasing application potential.

All of these animal-derived decellularized scaffolds are derived from abundant animal tissues and have a wide variety of uses, including organ transplantation, drug screening, and stem cell differentiation [67]. In the food industry, some animal-derived decellularized scaffolds have required edibility, high bio-compatibility and affinity, although the related studies are relatively lacking. Additionally, the environmental requirements for the comprehensive utilization of processing by-products are driving the research direction to become much more innovative, which shows greater development of animal-derived decellularized scaffolds.

## 5. The Methods and Application of Plant-Derived Decellularized Scaffolds

The animal-derived decellularized scaffolds exhibit some disadvantages, such as higher costs and immunogenicity. Therefore, because of the abundant resources of numerous plants on earth, plant-derived decellularized scaffolds are much more environmentally friendly, abundant and diverse.

### 5.1. Principles of Plant-Derived Decellularized Scaffolds

Plant-derived biomaterials have fewer residual nuclear components after the decellularization process [57]. The major component of the scaffolds is cellulose, which constitutes the plant cell walls. After long and sufficient research, cellulose has been proven to be an abundant biomaterial with high biocompatibility. It is low-cost and easy to produce, and has natural porosity for in vitro 3D mammalian cell culture [68]. Additionally, its tight and ordered structure cannot be degraded by enzymatic reactions [69], resulting in lower immunogenicity and tougher mechanical properties. At the same time, most of these plants have delicate veins and high surface areas that can provide a good support platform for cell adhesion, and even retain and transport water [70] to promote cell growth. Although plant leaves lack blood vessels, they can be pre-vascularized scaffolds to preserve vascularity, support the metabolic activities of mammalian cells [71] and deliver nutrients to tissues [72] after treatment. Moreover, decellularized scaffolds derived from edible fruits and vegetables can satisfy the requirements of being natural and edible. Overall, plant-derived decellularized scaffolds have complex and detailed structure, low cost, and high production, and can support cell adhesion, expansion, and alignment. Such scaffolds have been demonstrated to be feasible for mammalian cell growth [69]. 

### 5.2. Preparation Strategy for Plant-Derived Decellularized Scaffolds

The raw material of plant-derived decellularized scaffolds should be fresh and free from mechanical damage. Continuous chemical treatment is the main method for the preparation of plant-derived decellularized scaffolds [73]. Two decellularization methods have proven to be effective. One is a detergent-based method, which is similar to the methods for the preparation of animal-derived decellularized scaffolds, and the other is a detergent-free decellularization method [70]. Different treatments will produce decellularized scaffolds with different properties and have different effects on the activity of cells grown on the scaffold.

**Figure 3:**
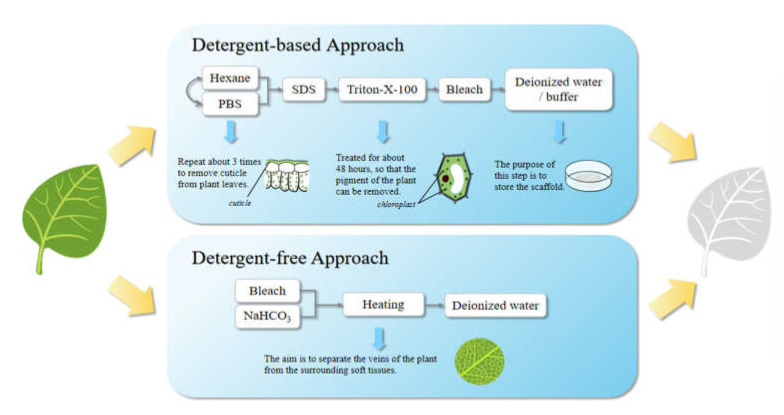
The methods for the preparation of plant-derived decellularized scaffolds.

The method of preparing plant-derived decellularized scaffolds is summarized in Figure 3. Among them, the first one is a detergent-based decellularization method. It can be subdivided into a perfusion method and immersion method, but they have no essential differences. First, leaf cuticles were removed by repeated treatment with hexane and phosphate buffer solution; secondly, phytopigments removal was achieved after the treatment with SDS water solution for about 5 days, 0.1% Triton-X-100 and 10% bleach solution for about 48 hours; thusly, the plant tissues will become white and transparent with clear veins. Finally, the scaffolds were immersed in deionized water or buffer solution and then stored there or lyophilized for storage. The steps of cuticles removal and phytopigments removal are not sequential. The second one is a detergent-free decellularization method. After the same pretreatment, the plant tissues were immersed in 5% bleach and a 3% sodium bicarbonate solution, and then heated and stirred in a fume hood. The plant veins can be separated from the surrounding soft tissues by such a treatment. When the temperature reaches 60 to 90 °C (The required temperature and treatment time depend on the plant species, sample sizes, etc.), the stirring speed should be gradually reduced to avoid damaging the tissue structure. Then, the scaffolds were placed in deionized water. Decellularized scaffolds can be evaluated by the roughness, porosity, surface area, hydrophilicity and mechanical properties of the scaffold surface [46]. 

### 5.3. Application in Cultured Meat Process

Plant scaffolds have great application prospects because of the species richness, the structural similarity to animal tissues, high reproducibility and biocompatibility, which are not only well-established but also the goal of researchers to explore and improve. In fact, more and more research is providing a strong basis for these theories. In order to efficiently prepare higher yielding and quality decellularized scaffolds, Harris et al. [73] proposed a rapid and sterile method for the preparation, the supercritical carbon dioxide (ScCO_2_) method. Combining ScCO_2_ with 2% peroxyacetic acid could remove plant tissue within 4 hours and preserve the plant microstructure. Spinach leaves, mint leaves, parsley stems and celery stems were treated to confirm the effectiveness of the method. Phan et al. [74] used deoxyribonuclease I (DNase I) to prepare decellularized scaffolds and then verified their ability in cell attachment, spreading and proliferation. Jones et al. [47] explored the potential application of decellularized spinach as an economical, efficient and environmentally friendly scaffold, which can provide an edible substrate for the growth of bovine satellite cells and pioneer innovative targeted directions for cultured meat. Through specific experiments on spinach, parsley, artemisia annua leaves and peanut hairy roots, Gershlak et al. [72] drew a conclusion with practical significance that the structural diversity of plants can be well adapted to the complexity of animal tissues, and therefore different plant tissues have their most suitable application directions for tissue engineering. Various plant-derived decellularized scaffolds can be selected for the in vitro culture of the same animal tissues. For the in vitro culture of skeletal muscle and bone tissue, Cheng et al. [49] used various vegetables, such as carrot, broccoli, cucumber, potato, apple, asparagus, green onion, leek and celery, to prepare decellularized scaffolds. The decellularized green onion scaffold has shown a greater ability to promote the arrangement and differentiation of skeletal muscle cells. By comparing and evaluating the decellularized scaffolds of apples, cauliflower, bell peppers, carrots, persimmons, and dates, Lee et al. [18] concluded that the decellularized apple scaffold with high biocompatibility and low costs can best promote the growth and differentiation of osteoblasts. Negrini et al. [50] showed that decellularized carrot scaffolds had greater potential than the decellularized scaffolds of apples and celery in promoting osteoclast adhesion, proliferation and differentiation. Salehi et al. preferred spinach leaves [46] and onion epidermis [67], while Aswathy et al. [52] explored the application of bamboo stems for this purpose. Worth mention, Allan et al. [53] studied grass blades. The surface of such a plant is full of grooves; therefore, it is a natural structure with directional and topographical features that can maintain cell viability, induce cell alignment, and support cell attachment and proliferation, and the differentiation of murine C2C12 myoblasts. This result suggested that some plant-derived scaffolds with special structures (e.g., repetitive grooves) may be more suitable for the alignment of muscle fibers [75]. Unlike most studies that focused on the edibility and degradability of scaffolds, Ong et al. [29] focused on the ability of scaffolds to improve the visual appearance of cell-cultured meat. For example, the jackfruit scaffold is more acceptable to consumers due to the oxidation of its internal natural polyphenols, which can induce flesh-like color and produce a rich marbling visual on meat cuts. 

Additionally, plant-derived decellularized scaffolds have also been the focus of the direction of organ regeneration, such as in vitro culture of vascular, myocardial and renal tubules [76]. While others have focused on applications beyond the cultivation of animal tissues in vitro. These studies showed a low association with the scope of in vitro cultured meat reviewed in this paper, and therefore were not further described here. The results reported above illustrate that plant-derived decellularized scaffolds have continued room for exploration and high research value. However, while the application potential of plant-derived decellularization is receiving increasing attention, Lacombe et al. [77] pointed out that compared with standard cell culture models, plant-derived decellularized scaffolds will alter cell behavior, which can affect cell morphology, bioactivity and the proliferation rate. The results also forewarn us that this technique is not yet mature enough to be placed into large-scale production and application. Thus, many studies are further needed to address various issues that may be confronted within the practical production environment.

## 6. Extensive Application Prospects and Current Shortcomings of Decellularized Scaffolds

Decellularized scaffolds, a biomaterial, were first used in the pharmaceutical industry including in organ regeneration and transplantation, drug discovery and in vivo drug delivery [78]. These biomaterials definitely offer great possibilities for the development of cellular agriculture and cultured meat. They not only demonstrate positive effects on addressing future animal protein shortages by producing in vitro cultured meat rapidly and massively, but also can reduce or avoid foodborne diseases spread by meat contaminated with parasites and pathogens and carbon emissions of the aquaculture industry, and provide environmental protection, etc. Notably, medicine, agriculture, and animal husbandry are actually developing conjointly. Continuing advances in agriculture and natural biomaterials have a crucial directing effect on the trend of tissue engineering and regenerative medicine [79]. Thus, the biotechnology of modern cellular agriculture has high research value far beyond itself.

However, the production of cultured meat by decellularized scaffolds has some drawbacks and is still not ready for industrial application. First of all, real meat consists of skeletal muscle, fat and connective tissue, etc. Skeletal muscle cells are adherent cells. Although decellularized scaffolds can provide a 3D structure for skeletal muscle cell growth, the way it controls the differentiation of skeletal muscle cells during their growth has not been explained yet. Fat is an essential flavor substance, and its tissue culture has relatively low technical difficulty. However, problems such as how to establish the muscle and fat structured co-culture, or how to assemble them after culturing remain to be addressed in the future [80]. Secondly, most of the current cultured meat obtained is finely minced and does not have the texture of real meat, which is unable to meet the consumers’ expectations and demands for meat quality macroscopically, and is also not accepted by consumers because its concept is too far ahead. Thirdly, natural decellularized scaffolds are not homogeneous and their structure cannot be artificially controlled or designed, so their quality is not stable enough and may even have safety problems because of the scaffold material itself. During the production of cultured meat in industry, a series of problems, such as the small culture scale, high costs and lack of automated equipment, are also present. 

Currently, the main sources of biological decellularized scaffolds include animals, plants and microorganisms. In the animal-derived decellularized scaffolds, decellularized mammalian tissues and organs occupy the largest proportion among them, whose components are mainly collagen, elastin, etc. Secondly, plant-derived decellularized scaffolds mainly contain grains, oilseed plants, herbaceous or Gramineae plants, algal, fruits and vegetables. The main component is cellulose among these plant-derived scaffolds. Lastly in the microorganisms, chitin was widely present in a variety of fungi [81], and is a natural material that is capable of forming nanofibers by a combination with polysaccharides, proteins and other substances [82]. Therefore, these biological decellularized scaffolds provide new insight to develop biomaterial because of their complex three-dimensional structure, antimicrobial properties, high biocompatibility and low immunogenicity. For example, Balasundari et al. [54] found that the decellularized scaffolds derived from *Agaricus bisporus* were able to induce osteogenic differentiation. Consequently, in the process of culturing decellularized scaffolds, searching for the appropriate and bioactive cultured material, which possess specific pro-growth and pro-differentiation functions, will continue to be a focus of future research.

## 7. Concluding Remarks

In summary, a systematic review of concepts, principles, preparation methods and research progress of animal-derived and plant-derived decellularized scaffolds has been presented. Scaffolds have great potential in the application of cultured meat due to their relatively large surface areas and high transport efficiency of substances. Biosource decellularized scaffolds are much more advanced than 3D-printed scaffolds because of high biocompatibility and excellent features in the adhesion, proliferation and differentiation of cells. Researchers have combined physical, chemical, and biological methods to constantly improve the preparation of scaffolds, increase decellularization efficacy, and explore their practical application values. Currently, animal-derived decellularized scaffolds have been more thoroughly studied, and some of them have reached the high standards requirement for clinical medicine. Nowadays, the majority of cultured meat was successfully obtained by growing on collagen or gelatin scaffolds [10]. In the plant-derived decellularized scaffolds, researchers have suggested the application value of plant-derived decellularized scaffolds for cultured meat because they are economical, efficient, and environmentally friendly. Additionally, most animal-derived decellularized scaffolds are used for the production of allogeneic tissues, while plant-derived decellularized scaffolds with various structures can be adapted to different and complex animal tissue cultures. In conclusion, both animal-derived and plant-derived decellularized scaffolds have been demonstrated to have the ability to be applied for culturing skeletal muscle, fat and connective tissue, which constitute the main components of edible meat. These findings have explained the feasibility and great future potential of industrial production of bio-decellularized scaffolds for cellular agriculture.

## Figures and Tables

**Table 1:** Summary of applied studies on the decellularized scaffolds in the food industry.

Decellularized Tissue	Decellularization Protocols	Application	Classification	Cell Type	References
Animal-derived Decellularized Scaffolds					
Porcine ligament	1% Triton X-100 and 200 U/mL DNase and 50 U/ml RNase	Skeletal tissues differentiation	Connective tissue	Human synovium derived mesenchymal stem cells (hSMSCs)	[32]
Porcine synovium	1% Triton X-100 and 200 U/mL DNase and 50 U/ml RNase	Skeletal tissues differentiation	Connective tissue	hSMSCs	[32]
Porcine subcutaneous	Digested with 0.05% trypsin for 16 h, underwent 48 h in 99.9% isopropanol, incubated with Benzonase digestion solution for 16 h and 99.9% isopropanol for 8 h, and disinfected with 0.1% peracetic acid in 4% ethanol	Promote morphological changes and cell differentiation	Fat	Human adipose stem cells (hASCs)	[33]
Human dermis fat	Digested in collagenase type I for 45 min at 37 °C	Promote cell adhesion, proliferation and differentiation	Connective tissue	hASCs	[34]
Porcine fat/adipose	1% Triton X-100 and 200 U/mL DNase and 50 U/mL RNase	Skeletal tissues differentiation	Connective tissue	hSMSCs	[32]
Porcine fat pad	1% Triton X-100 and 200 U/mL DNase and 50 U/mL RNase	Skeletal tissues differentiation	Connective tissue	hSMSCs	[32]
Fish scale	Incubated in 10 mM Tris-HCl buffer and 0.1% EDTA at 4 °C for 24 h and 0.1% SDS in Tris-HCl buffer at 4 °C for 3 days, and rinsed with 70% ethanol	Promote cell proliferation, increase osteogenic activity	Connective tissue	Human osteosarcoma cells (SaOS-2)	[35]
Cartilage	Sodium dodecyl sulfate, Triton X-100, ethylenediaminetetraacetic acid and Tris-Hydrochloride	Promote chondrogenic differentiation and proliferation	Connective tissue	Chondrocytes/stem cells	[36]
Porcine cartilage	1% Triton X-100 and 200 U/mL DNase and 50 U/ml RNase	Skeletal tissues differentiation	Connective tissue	hSMSCs	[32]
Sturgeon fish cartilage	Incubated in 1% SDS/PBS at 4 °C, and exposed to 0.1% EDTA/PBS, and digested in 1 U/mL DNase-I solution for 24 h, respectively	Induce matrix synthesis	Connective tissue	hASCs	[37]
Cartilage	Soaked in 1% Triton X-100 for 1d and 1% SDS	Induce the osteogenic differentiation of MSCs	Connective tissue	Mesenchymal stem cells (MSCs)	[38]
Porcine auricular cartilage	Washed in 10 mM Tris–HCl, 2 mM EDTA, 5 mM MgCl_2_, 100 mM DTT, 1% SDS, and 1% Triton-X100, incubated in PBS with 21 U/mL of hyaluronidase, and treated with DNase and RNase	Support cell attachment and growth	Connective tissue	Chondrocyte cells	[39]
Squid cranial cartilage	0.02% EDTA and 0.05 μg mL^−1^ trypsin solution	Promote chondrocyte migration, maintain its viability and spreading morphology	Connective tissue	Chondrocyte cells	[40]
Porcine meniscus	1% Triton X-100 and 200 U/mL DNase and 50 U/mL RNase	Skeletal tissues differentiation	Connective tissue	hSMSCs	[32]
Porcine bone	1% Triton X-100 and 200 U/mL DNase and 50 U/mL RNase	Skeletal tissues differentiation	Connective tissue	hSMSCs	[32]
Tendon	SDS, Triton X-100, trypsin, and freezing-thawing	Similar to natural tendons in bioactive components, collagen arrangement and biomechanical characteristics; recellularization and repair ability	Skeletal muscle	Tendon cells	[41]
Porcine tendon	1% Triton X-100 and 200 U/mL DNase and 50 U/mL RNase	Skeletal tissues differentiation	Connective tissue	hSMSCs	[32]
Equine-derived tendon	1% TBP followed by 1% PAA,1% TBP followed by 3% PAA, 1% TBP followed by 5% PAA, and 1% TBP	Promote osteogenic differentiation and tendon reconstruction	Skeletal muscle	Bone marrow mesenchymal stem cells (BMSCs)	[42]
Porcine muscle	1% Triton X-100 and 200 U/mL DNase and 50 U/mL RNase	Skeletal tissues differentiation	Connective tissue	hSMSCs	[32]
Rat muscle and dermis	Treated with 0.25% SDS and DNase I	Promote myocyte differentiation, reduce inflammation and fibrosis of the muscle	Skeletal muscle	Muscle spectrum cells	[43]
Porcine tibialis anterior skeletal muscle	Incubated in 1% SDS for 3d, washed with 50 U mL^−1^ DNase and 1 U mL^−1^ RNase in 10 mM Tris–HCl, treated with 0.5% Triton X-100 and Isopropanol for 1d, respectively	Enhance myogenic differentiation and maturation	Skeletal muscle	Myogenic cells	[44]
Mouse diaphragmatic extracellular matrix	Processed with three 4% sodium deoxycholate-2000 kU DNase-I treatment (DET) cycles	Promote proliferation and differentiation capability of muscle precursors, generate functional 3D skeletal muscle tissue constructs	Skeletal muscle	Muscle precursor cells	[45]
Plant-derived Decellularized Scaffolds					
Spinach	Rinsed in a normal hexane (98%) for 5 min, incubated in 10X SDS for 5 days at 25 °C, washed with 0.1% Triton-X100 in 10% sodium hypochlorite for 2 days	Promote osteogenic differentiation of stem cells in bone tissue engineering	Connective tissue	Bone marrow derived mesenchymal stem cells	[46]
Spinach	Rinsed in a normal hexane (98%) for 3 min, incubated in 1% SDS for 5 days, washed with 0.1% Triton-X100 and 10% concentrated bleach for 2 days	Provide an edible substrate for the growth of bovine satellite cells	Skeletal muscle	Primary bovine satellite cells	[47]
Onion	Rinsed in a normal hexane (98%) for 5 min, incubated in 10% SDS for 5 days at 25 °C, washed with 0.1% Triton-X100 in 10% sodium hypochlorite for 2 days	Promote the osteogenic differentiation of bone mesenchymal stem cells	Connective tissue	MSCs	[48]
Carrot	Submerged in 1% SDS and shaken at 70 rpm at 25 °C for 3 weeks, with the 1% SDS solution refreshed weekly	Promote the arrangement and differentiation of human and mouse skeletal muscle cells	Skeletal muscle	Mouse and human muscle cells	[49]
Carrot	Immersed 0.1% SDS for 48 h and washed in 100 mM CaCl_2_ for 24 h	Promote osteoclast adhesion, proliferation, and differentiation	Connective tissue	3T3-L1 preadipocytes, MC3T3-E1 Pre-osteoblasts and L929 cells	[50]
Broccoli	Submerged in 1% SDS and shaken at 70 rpm at 25 °C for 3 weeks, with the 1% SDS solution refreshed weekly	Promote the arrangement and differentiation of human and mouse skeletal muscle cells	Skeletal muscle	Mouse and human muscle cells	[49]
Cucumber	Submerged in 1% SDS and shaken at 70 rpm at 25 °C for 3 weeks, with the 1% SDS solution refreshed weekly	Promote the arrangement and differentiation of human and mouse skeletal muscle cells	Skeletal muscle	Mouse and human muscle cells	[49]
Potato	Submerged in 1% SDS and shaken at 70 rpm at 25 °C for 3 weeks, with the 1% SDS solution refreshed weekly	Promote cell arrangement and differentiation	Skeletal muscle	Mouse and human muscle cells	[49]
Apple	Submerged in 0.5% SDS for 48 h	Promote the growth and differentiation of osteoblasts	Connective tissue	Pluripotent stem cells	[18]
Broccoli florets	10% SDS, 3% Tween-20, and 10% bleach for 48 h	Promote cell adhesion	Connective tissue	Primary bovine satellite cells	[51]
Asparagus/ Green onion/Leek/Celery	Submerged in 1% SDS and shaken at 70 rpm at 25 °C for 3 weeks, with the 1% SDS solution refreshed weekly	Promote cell arrangement and differentiation	Skeletal muscle	Mouse and human muscle cells	[49]
Bamboo stems	(a) 10% SDS in 1% sodium hypochlorite (b) 1% Triton X-100 in 1% sodium hypochlorite (c) 10%SDS and 1% Triton X-100 in 1% sodium hypochlorite (d) 1% sodium hypochlorite	Promote cell adhesion, proliferation and osteogenic differentiation, and enhance bone tissue regeneration	Connective tissue	Mesenchymal stem cells	[52]
Grass blades	Agitated in 1% SDS, 1% Tween-20, and 10% bleach for 1 to 2 days	Maintain cell viability; induce cell alignment; support cell attachment, proliferation, and differentiation	Skeletal muscle	Murine C2C12 myoblasts	[53]
Jackfruit	Treated with 10% SDS for 5 days with gentle shaking, followed by washing in 0.1% Triton X-100 for 2 days	Promote cell adhesion and culture primary porcine myogenic cells with a rough surface	Skeletal muscle	Porcine myoblasts	[29]
Macrofungi-derived decellularized scaffolds					
Macrofungi *Agaricus bisporus*	Sodium Deoxycholate and Nucleases	Induce osteogenic differentiation	Connective tissue	Human mesenchymal stem cells (hMSCs)	[54]

## Data Availability

Not applicable.
